# Ruptured ovarian dermoid causing chemical peritonitis: a case report

**DOI:** 10.1093/bjrcr/uaaf004

**Published:** 2025-02-10

**Authors:** Vrinda Chandar, Joel Kevin Raj Samuel, Ajay Kumar Singh

**Affiliations:** Department of Radiology, Massachusetts General Hospital, Boston, MA 02114, United States; Department of Radiology, Massachusetts General Hospital, Boston, MA 02114, United States; Department of Radiology, Massachusetts General Hospital, Boston, MA 02114, United States

**Keywords:** Dermoid cyst, ovarian teratoma, chemical peritonitis, ovarian dermoid, dermoid rupture, spontaneously ruptured ovarian dermoid

## Abstract

Spontaneous rupture of ovarian dermoid cysts is uncommon. We describe a case of a 32-year-old female who presented to the emergency room with abdominal pain and distension. The patient was discovered to have a ruptured dermoid cyst which caused chemical peritonitis and was managed surgically.

## Clinical presentation

A 32-year-old nulliparous female presented to the emergency room with abdominal distension and pain for the last 2 days. The patient denied any history of vomiting, fever, or trauma. Past medical history was notable for intravenous substance abuse. At the time of admission, the patient was haemodynamically stable, afebrile, and normotensive, but tachycardic with a heart rate of 110 bpm and was somnolent with guarding and rebound tenderness. Blood investigations done at admission showed leucocytosis with a white blood cell count of 46 000 × 10^9^/L and elevated C-reactive protein (122 mg/L) and erythrocyte sedimentation rate (108 mm/h). A urine pregnancy test was negative. Differential considerations at this point were broad and included a tubo-ovarian abscess, appendicitis, or another intra-abdominal source of infection.

## Imaging findings

**Figure 1.**
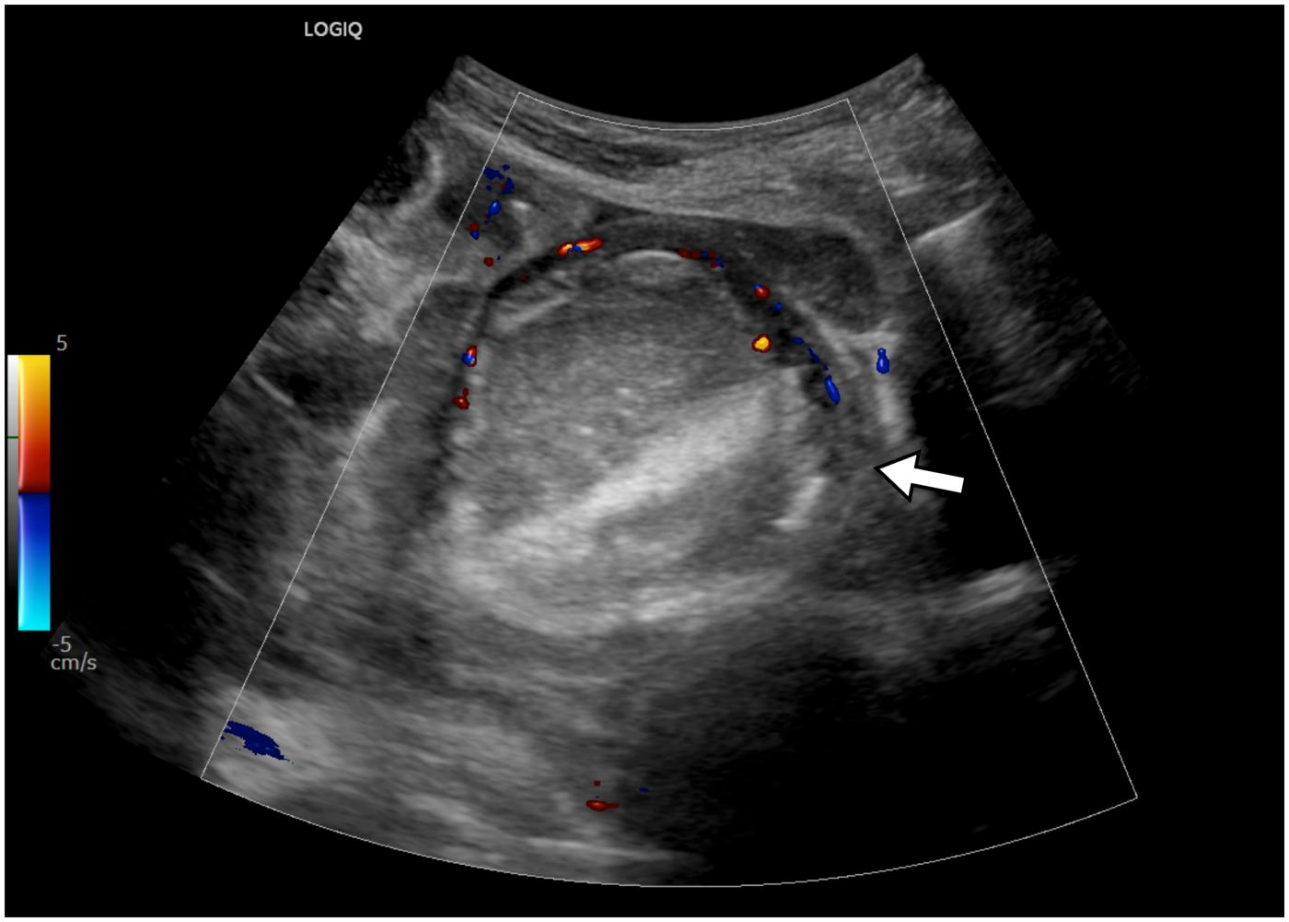
Ultrasonogram of the pelvis shows a well-defined, hyperechoic mass in the left adnexa, without internal vascularity. The left ovarian stroma is demonstrated at the periphery, showing vascularity on colour Doppler (arrow).

**Figure 2.**
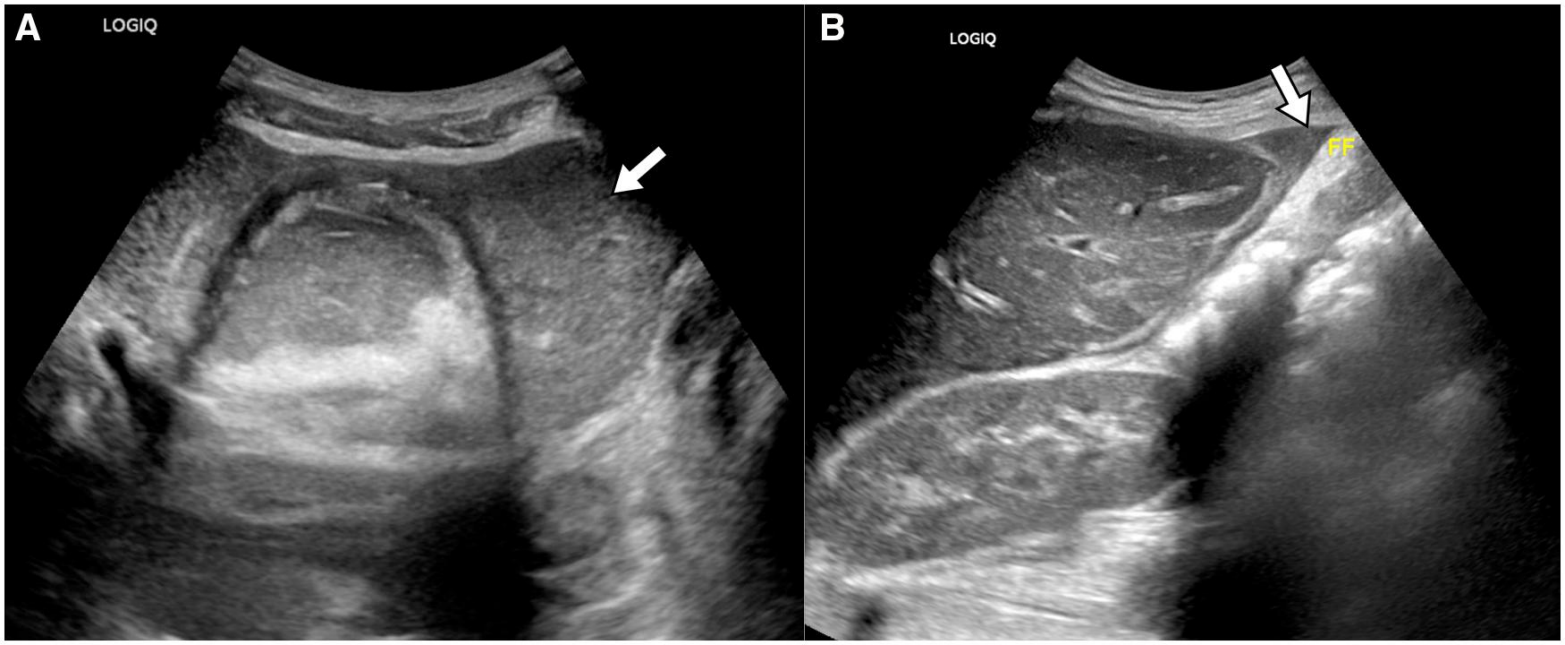
(A) Ultrasonogram of the pelvis shows echogenic fluid surrounding the left adnexal mass (arrow). (B) The echogenic fluid is also seen at the anterior surface of the liver (arrow). This echogenic fluid may represent blood or lipid laden fluid within the peritoneal cavity.

An ultrasound of the abdomen was done and demonstrated a hyperechoic mass lesion in the left adnexa, appearing to arise from the left ovary, without any internal vascularity (Figure 1). Surrounding the pelvic mass, there was echogenic free fluid, with swirling internal echogenicity, which extended up to the liver (Figure 2). This was interpreted as a ruptured ovarian cyst with hemoperitoneum.

**Figure 3.**
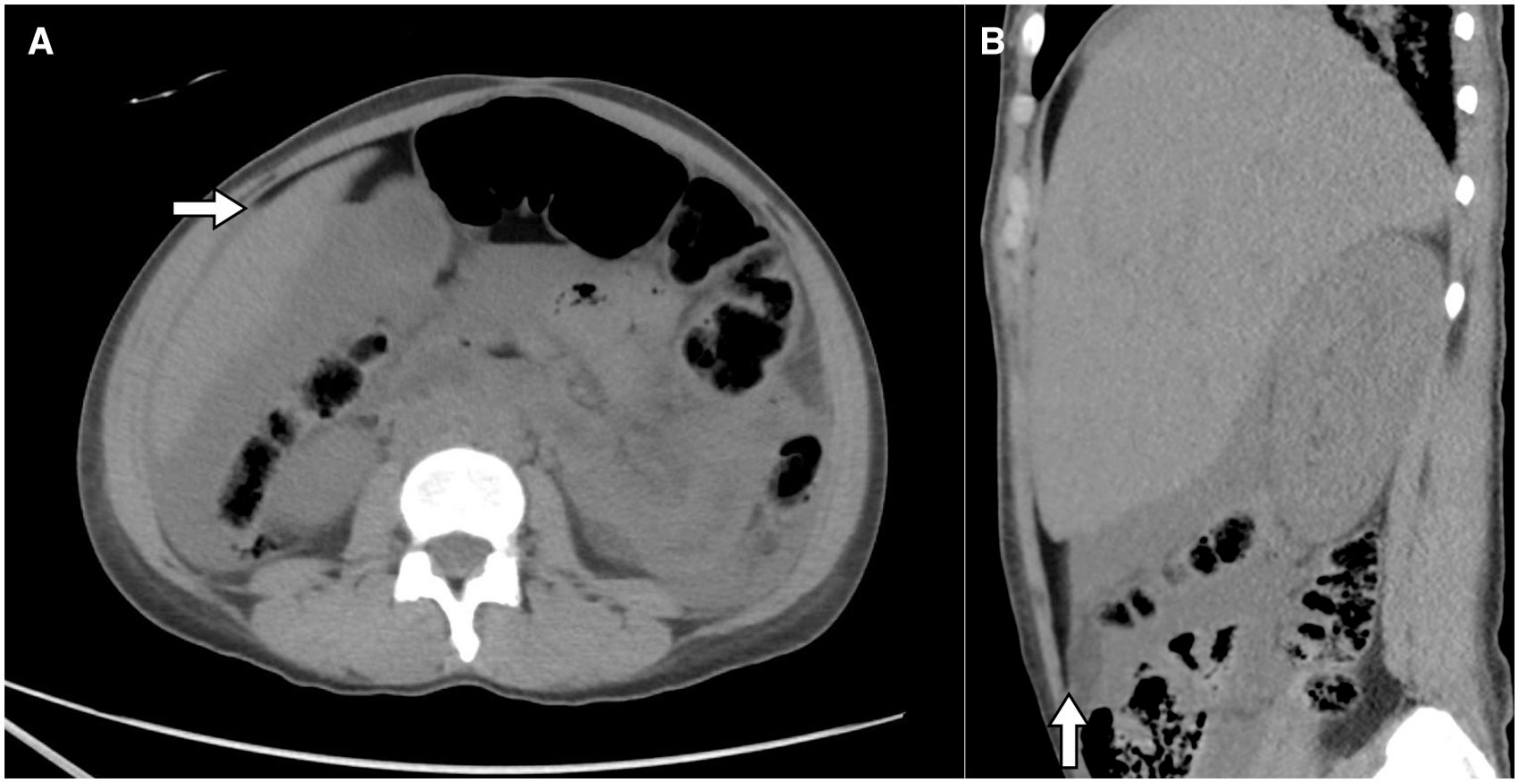
Axial CT section at the level of the upper abdomen (A) and right parasagittal reformation (B) shows fluid attenuation in the dependent areas, and fat attenuation in the non-dependent areas, forming a fat- fluid level. The anterior margin of the liver (arrow in (A)) is surrounded by the fat attenuation fluid.

**Figure 4.**
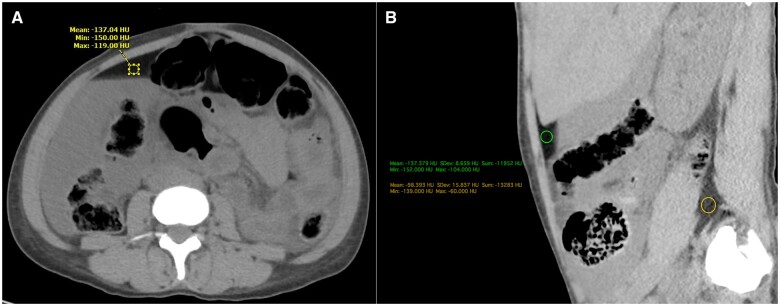
(A) Axial CT section at the level of the mid abdomen shows dependent high attenuation fluid, filling the right paracolic gutter, and non-dependent low attenuation fat towards the anterior abdominal wall, forming a fat-fluid level. A region of interest (ROI) in the non-dependent fluid, shows attenuation value of −137 Hounsfield Units (HU), suggestive of fat. (B)This non-dependent fluid has a lower attenuation value than the mesenteric or retroperitoneal fat (−98 HU). This fluid was consistent with the lipid laden peritoneal fluid that was found on laparotomy.

**Figure 5.**
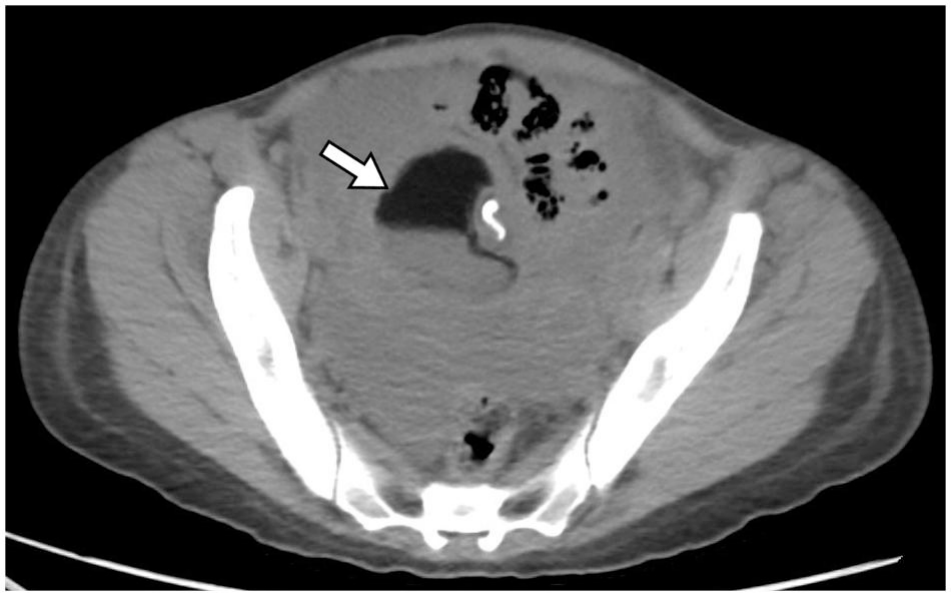
Axial CT section at the level of the pelvis shows a mass in the right adnexa with internal fat, fluid, and calcifications, consistent with a dermoid cyst. There is an internal fat-fluid level. There is also hemorrhagic fluid surrounding the cyst in the pelvis.

An unenhanced CT of the abdomen was done, in view of poor renal function. The CT demonstrated a large amount of intraperitoneal free fluid, with dependent higher attenuation fluid, representing hemoperitoneum. Non-dependently, there was also low attenuation fluid forming a fluid level with the haemorrhagic fluid (Figure 3). A region of interest within the low attenuation fluid confirmed an attenuation value consistent with fat density (Figure 4). This non-dependent fluid had a lower attenuation than mesenteric fat and was interpreted to represent lipid laden fluid. In the pelvis, there was a mass with internal fat, fluid, and calcifications, consistent with an ovarian dermoid cyst (Figure 5). This constellation of imaging findings was interpreted to represent a ruptured dermoid cyst with a large volume of intraperitoneal free fluid and fat.

## Treatment and outcome

Surgery was indicated and a diagnostic laparoscopy was performed. Intraperitoneally, there were copious amounts of lipid laden fluid with hair, consistent with the contents of a ruptured dermoid cyst. The bowel was also oedematous and coated with phlegmonous and fibrinous material. A right salphingo-oopherectomy was performed with copious irrigation and pelvic washout.

Cytology of the peritoneal fluid was negative for malignant tumours but showed fat consistent with a ruptured dermoid. Histopathological examination of the right ovary confirmed the findings of a mature cystic teratoma, with areas of skin with sebaceous glands, cartilage, and intestinal epithelium. The postoperative course was complicated by multifocal pneumonia, acute kidney injury, and pre-existing cellulitis of the right hand. Empirical antibiotics were transitioned to linezolid, cefepime, and metronidazole based on culture analysis. The patient made a full recovery with intensive care and was discharged home.

## Discussion

Mature cystic teratomas, or dermoid cysts are a common ovarian neoplasm, characterized by elements from all 3 germ cell lines, the ectoderm (skin, hair), mesoderm (fat, calcifications), and endoderm (ciliated or columnar epithelium).^1^ They are slow growing, benign tumours which are commonly asymptomatic, which may delay diagnosis and treatment.^2^

On ultrasound, the most common finding is a cystic mass, with a dense, shadowing focus (Rokitansky nodule), or an echogenic region of fat, or thin, echogenic bands due to hair within the cyst. On CT and MR, the diagnosis is simple due to the sensitivity for fat. CT may detect calcifications, and MR may detect high T1 signal within the sebaceous component.^1^ A fat fluid level, or floating sebaceous or keratinous material within the cystic fluid (described as a “boba sign”^3^) may also be seen.

Rupture of dermoid cysts is relatively rare due to their thick capsule,^2^ with a reported frequency as low as 0.3%-2%.^4^ The most common causes of rupture appear to be torsion and pregnancy, followed by trauma. Pregnancy may lead to torsion, likely due to the changes in position of the ovaries and increasing vascularity.^5^ This case report is unusual in that a clear precipitating factor for dermoid cyst rupture could not be determined. The size of the dermoid cyst seems to correlate to a higher risk of rupture. Eighty percent of ovarian cysts ruptured when they were above 60 mm. Comparably, the rupture rate of cysts smaller than 60 mm was 51%.^6^

Chemical peritonitis is a relatively uncommon complication of a ruptured dermoid cysts. The clinical presentation of peritonitis may be acute or chronic. Acute peritonitis is seen after the leak of a large volume of sebaceous material due to a sudden cyst rupture, causing acute abdominal symptoms, as in this patient. Chronic peritonitis is due to slow exudation of cyst contents through a small tear and presents with insidious onset of symptoms.^4^ Dermoid cysts, when they have not ruptured, are usually managed conservatively as they are slow growing.^7^ Surgical management may be indicated for uncomplicated cysts that are greater than 10 cm, and for post-menopausal patients due to the risk of malignant transformation.^5^

## Learning points

- An intra-peritoneal fat fluid level is a characteristic feature of a ruptured dermoid cyst.

## Informed consent statement

Written informed consent was obtained from the patient for publication of this case report, including accompanying images.

## References

[1] Outwater EK , Siegelman ES, Hunt JL. Ovarian teratomas: tumor types and imaging characteristics. Radiographics. 2001;21:475-490. 11259710 10.1148/radiographics.21.2.g01mr09475

[2] Mazhoud I , Skhiri W, Hafsa C, Toumi D, Maatouk M, Ben Salem A. Ruptured mature ovarian teratoma: a case report. Int J Surg Case Rep. 2023;102:107788. 36516595 10.1016/j.ijscr.2022.107788 PMC9768295

[3] Chang AY , Sun DC, Ohliger MA, Abuzahriyeh T, Choi HH. Boba sign: a novel sign for floating balls within a mature cystic teratoma. Abdom Radiol (NY). 2020;45:2931-2933. 32656580 10.1007/s00261-020-02647-8

[4] Takeda A , Koike W. Clinical characteristics and laparoscopic surgical outcomes of ovarian dermoid cysts complicated by spontaneous rupture: nine cases and a literature review. J Int Med Res. 2023;51:3000605231171023. 10.1177/03000605231171023 37138472 PMC10161322

[5] Li RY , Nikam Y, Kapurubandara S. Spontaneously ruptured dermoid cysts and their potential complications: a review of the literature with a case report. Case Rep Obstet Gynecol. 2020;2020:e6591280. https://www.hindawi.com/journals/criog/2020/6591280/10.1155/2020/6591280 PMC7150697 32292616

[6] Agboola AA , Uddin K, Taj S, et al Dermoid cyst spillage resulting in chemical peritonitis: a case report and literature review. Cureus. 2022;14:e29151. 36258939 10.7759/cureus.29151 PMC9562604

[7] Hoo WL , Yazbek J, Holland T, Mavrelos D, Tong ENC, Jurkovic D. Expectant management of ultrasonically diagnosed ovarian dermoid cysts: is it possible to predict outcome? Ultrasound Obstet Gynecol. 2010;36:235-240. 20201114 10.1002/uog.7610

